# *trans*-Bis[4-amino-3,5-bis­(2-pyrid­yl)-4*H*-1,2,4-triazole-κ*N* ^3^]diaqua­cobalt(II) bis­(3-carb­oxy-5-nitro­benzoate)

**DOI:** 10.1107/S1600536811035446

**Published:** 2011-09-14

**Authors:** Xi Wang, Chun-Fu Shao, Cheng-Peng Li

**Affiliations:** a College of Chemistry, Tianjin Key Laboratory of Structure and Performance for Functional Molecules, Tianjin Normal University, Tianjin 300387, People’s Republic of China

## Abstract

The title complex, [Co(C_12_H_10_N_6_)_2_(H_2_O)_2_](C_8_H_4_NO_6_)_2_, is composed of a mononuclear cobalt(II) cation and two 3-carb­oxy-5-nitro­benzoate anions for charge balance. In the cation, the Co^II^ atom is six-coordinated in a distorted octa­hedral geometry. It bonds to two O atoms of two water mol­ecules, and two pairs of N atoms from two 4-amino-3,5-bis­(2-pyrid­yl)-4*H*-1,2,4-triazole mol­ecules, which behave as bidentate chelating ligands. There are intra­molecular N—H⋯N hydrogen bonds in the cation. In the crystal, there are a number of inter­molecular N—H⋯O and O—H⋯O hydrogen bonds, as well as inter­molecular π–π stacking inter­actions [centroid–centroid distances = 3.657 (2) and 3.847 (2) Å], that link the mol­ecules into two-dimensional networks lying parallel to the *ab* plane. The presence of C—H⋯O inter­actions leads to the formation of a three-dimensional network.

## Related literature

For background information on triazole derivatives, see: Klingele *et al.* (2009 ▶); Shao *et al.* (2004 ▶); Huang *et al.* (2011 ▶). For the coordination systems of related pyridyl-substitued triazole ligands, see: Du *et al.* (2007 ▶, 2008 ▶); He *et al.* (2010 ▶); Li *et al.* (2010 ▶). For some examples of the coordination complexes of the title (4-amino-3,5-bis­(2-pyrid­yl)-4*H*-1,2,4-triazole (2-bpt) ligand, see: Guo *et al.* (2011 ▶).
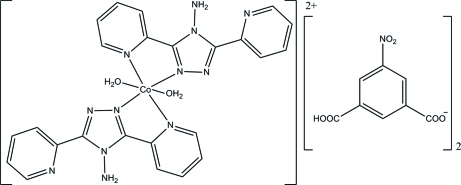


## Experimental

### 

#### Crystal data

[Co(C_12_H_10_N_6_)_2_(H_2_O)_2_](C_8_H_4_NO_6_)_2_
*M* *_r_* = 991.73
Triclinic, 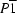

*a* = 7.589 (3) Å
*b* = 16.104 (6) Å
*c* = 18.256 (6) Å
α = 74.739 (6)°
β = 86.435 (6)°
γ = 88.619 (6)°
*V* = 2148.4 (13) Å^3^
*Z* = 2
Mo *K*α radiation
μ = 0.49 mm^−1^
*T* = 296 K
0.28 × 0.20 × 0.10 mm

#### Data collection

Bruker SMART CCD area-detector diffractometer
Absorption correction: multi-scan (*SADABS*; Sheldrick, 1996 ▶) *T*_min_ = 0.876, *T*_max_ = 0.953
11079 measured reflections
7532 independent reflections
5560 reflections with *I* > 2σ(*I*)
*R*_int_ = 0.020

#### Refinement

*R*[*F* ^2^ > 2σ(*F* ^2^)] = 0.039
*wR*(*F* ^2^) = 0.098
*S* = 1.03
7532 reflections
624 parameters
H-atom parameters constrained
Δρ_max_ = 0.19 e Å^−3^
Δρ_min_ = −0.28 e Å^−3^

### 

Data collection: *SMART* (Bruker, 2007 ▶); cell refinement: *SAINT* (Bruker, 2007 ▶); data reduction: *SAINT*; program(s) used to solve structure: *SHELXS97* (Sheldrick, 2008 ▶); program(s) used to refine structure: *SHELXL97* (Sheldrick, 2008 ▶); molecular graphics: *DIAMOND* (Brandenburg, 1999 ▶); software used to prepare material for publication: *SHELXTL* (Sheldrick, 2008 ▶).

## Supplementary Material

Crystal structure: contains datablock(s) I, global. DOI: 10.1107/S1600536811035446/su2310sup1.cif

Structure factors: contains datablock(s) I. DOI: 10.1107/S1600536811035446/su2310Isup2.hkl

Additional supplementary materials:  crystallographic information; 3D view; checkCIF report

## Figures and Tables

**Table 1:** Hydrogen-bond geometry (Å, °)

*D*—H⋯*A*	*D*—H	H⋯*A*	*D*⋯*A*	*D*—H⋯*A*
N5—H5*A*⋯N6	0.89	2.23	2.885 (3)	130
N11—H11*A*⋯N12	0.89	2.21	2.859 (3)	130
O1—H1*A*⋯O9^i^	0.85	1.85	2.674 (3)	163
O1—H1*B*⋯O5^ii^	0.85	1.87	2.710 (3)	168
O2—H2*A*⋯O5^iii^	0.85	1.89	2.712 (2)	162
O2—H2*B*⋯O9	0.85	1.86	2.681 (3)	163
O4—H4⋯O10^iv^	0.82	1.73	2.545 (3)	172
N5—H5*B*⋯O10	0.89	2.36	3.065 (3)	136
N5—H5*B*⋯O3^iv^	0.89	2.51	3.234 (3)	139
N11—H11*B*⋯O6^iii^	0.89	2.32	3.057 (3)	140
N11—H11*B*⋯O13^v^	0.89	2.56	3.252 (3)	135
O14—H14⋯O6^vi^	0.82	1.77	2.580 (3)	170
C3—H3⋯O5^vii^	0.93	2.47	3.402 (4)	176
C9—H9⋯O11^iv^	0.93	2.49	3.249 (5)	139
C14—H14*A*⋯O6^viii^	0.93	2.49	3.403 (4)	166
C21—H21⋯O7^ix^	0.93	2.48	3.259 (4)	142
C28—H28⋯O13^vi^	0.93	2.56	3.490 (4)	175

Symmetry codes: (i) 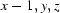; (ii) 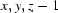; (iii) 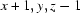; (iv) 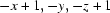; (v) 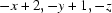; (vi) 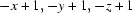; (vii) 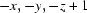; (viii) 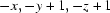; (ix) 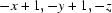.

**Fig. 1.**
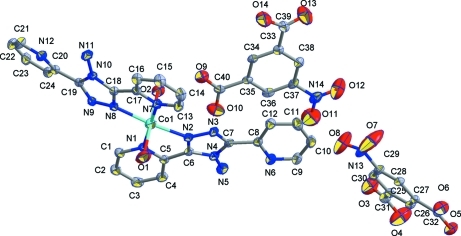
Molecular structure of the title compound showing the numbering scheme, and displacement ellipsoids drawn at the 50% probability level [H-atoms have been omitted for clarity].

**Fig. 2.**
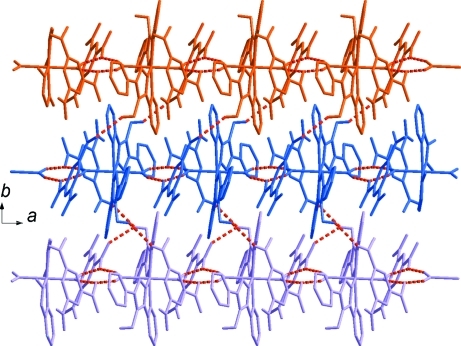
A view along the c-axis of the two-dimensional network in the crystal of the title compound, formed via N–H···O and O–H···O hydrogen bonds (red dashed lines; see Table 1 for details).

## supplementary crystallographic information

### Comment

1,2,4-triazole and its derivatives are an important class of ligands used as building tectons in constructing coordination complexes (Klingele *et al.*, 2009; Shao *et al.*, 2004; Huang *et al.*, 2011). In particular, pyridyl-decorated triazole ligands are extensively studied, for example 4-amino-3,5-bis(4-pyridyl)-4*H*-1,2,4-triazole and 4-amino-3,5-bis(3-pyridyl)-4*H*-1,2,4-triazole, by us (Du *et al.*, 2007; 2008) and other groups (He *et al.*, 2010; Li, *et al.* 2010). In the published articles, such series of ligands successfully behave as not only the extended exo-bidentate linkers with the terminal pyridyl groups but also as the promoter of a coordinative cycle with the central triazole upon metalation (Li, *et al.* 2010). Toward this direction, the analogous ligand 4-amino-3,5-bis(2-pyridyl)-4*H*-1,2,4-triazole (2-bpt) exhibits different conformations and coordination modes, and has received our attention (Guo *et al.* 2011). In continuation of our work in this field we report on the crystal structure of the title cobalt(II) complex.

The molecular structure of title complex is illustrated in Fig. 1. In the cation [Co(2-bpt)_2_(H_2_O)_2_]^2+^, the 2-bpt ligands show a bidentate mode, bonding to atom Co1 through atoms N1, N2 and N7, N8, in a *trans* arrangement. The Co1 atom is also coordinated to a pair of water molecules through the O atoms O1 and O2. The cobalt atom shows a distorted octahedral coordination sphere. The two 3-carboxy-5-nitrobenzoates are isolated in a discrete mode for charge balance. Within the cation [Co(2-bpt)_2_(H_2_O)_2_]^2+^, there are two intramolecular N–H···N bonds involving the amino and the 2-pyridyl groups in the 2-bpt ligands (Table 1).

In the crystal, intermolecular O–H···O hydrogen bonds between the water ligand and discrete 3-carboxy-5-nitrobenzoates interlink these molecules into a one-dimensional chain motif. Furthermore, adjacent chains are interconnected by N–H···O hydrogen bonds between the 2-bpt and 3-carboxy-5-nitrobenzoate, and O–H···O hydrogen bonds between the 3-carboxy-5-nitrobenzoates, to form extended two-dimensional networks (Table 1 and Fig. 2). In addition, a number of *π*···*π* stacking interactions, involving symmetry related coordinated pyridine rings, are found to further reinforce the two-dimensional supramolecular pattern [Cg1···Cg1^i^ 3.847 (2) Å, perpendicular separation 3.534 (1) Å, with slippage of 1.519 Å; Cg2···Cg2^i^ 3.657 (2) Å, perpendicular separation 3.495 (1) Å, slippage 1.077 Å; where Cg1 is the centroid of ring (N1,C1-C5); Cg2 is the centroid of ring (N7,C13-C17); symmetry code: 1-x, -y, -z]. There are also a number of C-H···O interactions present linking the two-dimensional networks to form a three-dimensional structure (Tabel 1).

### Experimental

A mixture of 4-amino-3,5-bis(2-pyridyl)-1,2,4-triazole (2-bpt; 23.8 mg, 0.1 mmol), 5-nitroisophthalic acid (21.1 mg, 0.1 mmol), Co(NO_3_)_2_ (18.3 mg, 0.1 mmol) in water (10 ml) was sealed in a Teflon-lined stainless steel vessel (20 ml). It was heated to 373 K in 24 h and then gradually cooled to room temperature at a rate of 5 °C/h. Red block-like crystals, suitable for X-ray analysis, were obtained. Anal. Calc. for C_40_H_32_CoN_14_O_14_: C, 48.44; H, 3.25; N, 19.77%. Found: C, 48.32; H, 3.04; N, 19.85%.

### Refinement

All the H atoms were initially located in a difference Fourier map. The water and NH_2_ H atoms were then refined as riding atoms with *U*_iso_(H) = 1.5*U*_eq_(O,N). The hydroxyl and C-bound H atoms were included in calculated positions and treated as riding atoms: O-H = 0.85 Å, N-H = 0.89 Å, and C-H = 0.93 Å, with *U*_iso_(H) = k × *U*_eq_ (O,N,C), where k = 1.5 for OH and NH_2_ H atoms, and k = 1.2 for C-bound H-atoms.

### Crystal data

**Table:**

[Co(C_12_H_10_N_6_)_2_(H_2_O)_2_](C_8_H_4_NO_6_)_2_	*Z* = 2
*M**_r_* = 991.73	*F*(000) = 1018
Triclinic, *P*1	*D*_x_ = 1.533 Mg m^−^^3^
Hall symbol: -P 1	Mo *K*α radiation, λ = 0.71073 Å
*a* = 7.589 (3) Å	Cell parameters from 2949 reflections
*b* = 16.104 (6) Å	θ = 2.3–27.8°
*c* = 18.256 (6) Å	µ = 0.49 mm^−^^1^
α = 74.739 (6)°	*T* = 296 K
β = 86.435 (6)°	Block, red
γ = 88.619 (6)°	0.28 × 0.20 × 0.10 mm
*V* = 2148.4 (13) Å^3^	

### Data collection

**Table:**

Bruker SMART CCD area-detector diffractometer	7532 independent reflections
Radiation source: fine-focus sealed tube	5560 reflections with *I* > 2σ(*I*)
graphite	*R*_int_ = 0.020
φ and ω scans	θ_max_ = 25.0°, θ_min_ = 1.5°
Absorption correction: multi-scan (*SADABS*; Sheldrick, 1996)	*h* = −9→9
*T*_min_ = 0.876, *T*_max_ = 0.953	*k* = −19→14
11079 measured reflections	*l* = −21→16

### Refinement

**Table:**

Refinement on *F*^2^	Primary atom site location: structure-invariant direct methods
Least-squares matrix: full	Secondary atom site location: difference Fourier map
*R*[*F*^2^ > 2σ(*F*^2^)] = 0.039	Hydrogen site location: inferred from neighbouring sites
*wR*(*F*^2^) = 0.098	H-atom parameters constrained
*S* = 1.03	*w* = 1/[σ^2^(*F*_o_^2^) + (0.0395*P*)^2^ + 0.4823*P*] where *P* = (*F*_o_^2^ + 2*F*_c_^2^)/3
7532 reflections	(Δ/σ)_max_ = 0.001
624 parameters	Δρ_max_ = 0.19 e Å^−^^3^
0 restraints	Δρ_min_ = −0.28 e Å^−^^3^

### Special details

**Table:**

Geometry. All e.s.d.'s (except the e.s.d. in the dihedral angle between two l.s. planes) are estimated using the full covariance matrix. The cell e.s.d.'s are taken into account individually in the estimation of e.s.d.'s in distances, angles and torsion angles; correlations between e.s.d.'s in cell parameters are only used when they are defined by crystal symmetry. An approximate (isotropic) treatment of cell e.s.d.'s is used for estimating e.s.d.'s involving l.s. planes.
Refinement. Refinement of *F*^2^ against ALL reflections. The weighted *R*-factor *wR* and goodness of fit *S* are based on *F*^2^, conventional *R*-factors *R* are based on *F*, with *F* set to zero for negative *F*^2^. The threshold expression of *F*^2^ > σ(*F*^2^) is used only for calculating *R*-factors(gt) *etc*. and is not relevant to the choice of reflections for refinement. *R*-factors based on *F*^2^ are statistically about twice as large as those based on *F*, and *R*- factors based on ALL data will be even larger.

### Fractional atomic coordinates and isotropic or equivalent isotropic displacement parameters (Å^2^)

**Table:**

	*x*	*y*	*z*	*U*_iso_*/*U*_eq_	
Co1	0.32405 (4)	0.25256 (2)	0.003809 (17)	0.03888 (11)	
O1	0.0507 (2)	0.25767 (13)	0.00518 (10)	0.0705 (6)	
H1A	−0.0100	0.2350	0.0460	0.106*	
H1B	−0.0016	0.2660	−0.0360	0.106*	
O2	0.5949 (2)	0.24725 (11)	0.00307 (9)	0.0518 (5)	
H2A	0.6592	0.2653	−0.0376	0.078*	
H2B	0.6501	0.2252	0.0428	0.078*	
O3	0.3001 (3)	0.09926 (13)	0.66872 (12)	0.0740 (6)	
O4	0.1421 (4)	0.06414 (13)	0.77892 (13)	0.0909 (8)	
H4	0.1844	0.0163	0.7811	0.136*	
O5	−0.1561 (2)	0.27405 (11)	0.88632 (9)	0.0490 (4)	
O6	−0.1783 (2)	0.41069 (11)	0.82107 (10)	0.0549 (5)	
O7	0.1480 (5)	0.49569 (17)	0.57118 (15)	0.1329 (13)	
O8	0.2199 (4)	0.40024 (17)	0.51197 (12)	0.1040 (9)	
O9	0.8068 (2)	0.20782 (13)	0.11969 (10)	0.0588 (5)	
O10	0.7215 (3)	0.08548 (11)	0.20111 (12)	0.0630 (5)	
O11	0.8256 (4)	0.04771 (19)	0.47188 (14)	0.1199 (11)	
O12	0.9545 (4)	0.14525 (16)	0.50898 (13)	0.0921 (8)	
O13	1.1457 (3)	0.41543 (12)	0.32180 (12)	0.0784 (7)	
O14	1.0360 (3)	0.43935 (12)	0.20838 (11)	0.0710 (6)	
H14	1.0747	0.4877	0.2033	0.106*	
N1	0.3044 (3)	0.11527 (13)	0.02363 (11)	0.0445 (5)	
N2	0.3197 (3)	0.20794 (12)	0.12305 (11)	0.0403 (5)	
N3	0.3485 (3)	0.24343 (13)	0.18211 (11)	0.0430 (5)	
N4	0.3396 (3)	0.10213 (12)	0.22488 (11)	0.0425 (5)	
N5	0.3586 (3)	0.01656 (13)	0.27045 (12)	0.0587 (6)	
H5A	0.3185	0.0197	0.3165	0.088*	
H5B	0.4753	0.0095	0.2701	0.088*	
N6	0.3598 (3)	0.11979 (15)	0.37782 (12)	0.0578 (6)	
N7	0.3113 (3)	0.39029 (13)	−0.01663 (11)	0.0435 (5)	
N8	0.3581 (3)	0.29661 (12)	−0.11590 (11)	0.0405 (5)	
N9	0.4189 (3)	0.25979 (12)	−0.17272 (11)	0.0419 (5)	
N10	0.4397 (3)	0.40093 (12)	−0.21353 (11)	0.0417 (5)	
N11	0.4862 (3)	0.48528 (13)	−0.25670 (12)	0.0574 (6)	
H11A	0.4741	0.4837	−0.3045	0.086*	
H11B	0.6006	0.4887	−0.2499	0.086*	
N12	0.5556 (3)	0.38292 (15)	−0.36183 (12)	0.0589 (6)	
N13	0.1622 (4)	0.42141 (19)	0.56753 (15)	0.0786 (8)	
N14	0.8956 (4)	0.11715 (18)	0.45993 (15)	0.0692 (7)	
C1	0.2857 (4)	0.07359 (17)	−0.02980 (16)	0.0555 (7)	
H1	0.2917	0.1051	−0.0805	0.067*	
C2	0.2577 (4)	−0.01403 (18)	−0.01325 (17)	0.0629 (8)	
H2	0.2468	−0.0412	−0.0518	0.075*	
C3	0.2464 (4)	−0.05991 (19)	0.06188 (18)	0.0680 (9)	
H3	0.2267	−0.1189	0.0748	0.082*	
C4	0.2644 (4)	−0.01838 (17)	0.11821 (16)	0.0583 (8)	
H4A	0.2565	−0.0488	0.1692	0.070*	
C5	0.2940 (3)	0.06877 (16)	0.09748 (14)	0.0427 (6)	
C6	0.3153 (3)	0.12378 (15)	0.14923 (13)	0.0399 (6)	
C7	0.3618 (3)	0.17853 (16)	0.24301 (13)	0.0408 (6)	
C8	0.3936 (3)	0.18816 (17)	0.31903 (14)	0.0443 (6)	
C9	0.3847 (4)	0.1292 (2)	0.44718 (16)	0.0693 (9)	
H9	0.3638	0.0822	0.4888	0.083*	
C10	0.4392 (4)	0.2042 (2)	0.46033 (17)	0.0708 (9)	
H10	0.4538	0.2077	0.5096	0.085*	
C11	0.4718 (4)	0.2738 (2)	0.39973 (18)	0.0692 (9)	
H11	0.5076	0.3256	0.4072	0.083*	
C12	0.4506 (4)	0.26606 (18)	0.32698 (16)	0.0546 (7)	
H12	0.4740	0.3120	0.2847	0.066*	
C13	0.2701 (4)	0.43301 (18)	0.03589 (16)	0.0558 (7)	
H13	0.2541	0.4018	0.0865	0.067*	
C14	0.2502 (4)	0.52099 (18)	0.01830 (17)	0.0616 (8)	
H14A	0.2224	0.5487	0.0563	0.074*	
C15	0.2721 (4)	0.56709 (18)	−0.05627 (17)	0.0629 (8)	
H15	0.2591	0.6267	−0.0695	0.075*	
C16	0.3139 (4)	0.52431 (16)	−0.11160 (16)	0.0544 (7)	
H16	0.3289	0.5545	−0.1625	0.065*	
C17	0.3327 (3)	0.43628 (15)	−0.08981 (14)	0.0410 (6)	
C18	0.3730 (3)	0.38062 (15)	−0.14064 (13)	0.0397 (6)	
C19	0.4689 (3)	0.32364 (15)	−0.23130 (13)	0.0396 (6)	
C20	0.5518 (3)	0.31336 (17)	−0.30288 (14)	0.0451 (6)	
C21	0.6397 (4)	0.3751 (2)	−0.42676 (17)	0.0722 (9)	
H21	0.6454	0.4230	−0.4685	0.087*	
C22	0.7174 (4)	0.3000 (2)	−0.43443 (18)	0.0730 (9)	
H22	0.7764	0.2976	−0.4800	0.088*	
C23	0.7071 (4)	0.2286 (2)	−0.37412 (17)	0.0649 (8)	
H23	0.7565	0.1767	−0.3786	0.078*	
C24	0.6227 (4)	0.23449 (18)	−0.30664 (15)	0.0530 (7)	
H24	0.6135	0.1868	−0.2648	0.064*	
C25	0.1314 (3)	0.20759 (15)	0.70810 (14)	0.0436 (6)	
C26	0.0370 (3)	0.22969 (15)	0.76796 (14)	0.0412 (6)	
H26	0.0116	0.1874	0.8128	0.049*	
C27	−0.0203 (3)	0.31364 (15)	0.76225 (13)	0.0362 (5)	
C28	0.0197 (3)	0.37651 (16)	0.69598 (14)	0.0450 (6)	
H28	−0.0162	0.4332	0.6911	0.054*	
C29	0.1143 (4)	0.35339 (17)	0.63703 (14)	0.0489 (7)	
C30	0.1688 (3)	0.27040 (17)	0.64131 (15)	0.0493 (7)	
H30	0.2295	0.2567	0.6002	0.059*	
C31	0.1993 (4)	0.11865 (17)	0.71484 (17)	0.0548 (7)	
C32	−0.1256 (3)	0.33493 (16)	0.82824 (14)	0.0389 (6)	
C33	0.9871 (3)	0.30328 (15)	0.29271 (14)	0.0444 (6)	
C34	0.9275 (3)	0.27180 (15)	0.23500 (14)	0.0432 (6)	
H34	0.9331	0.3068	0.1855	0.052*	
C35	0.8596 (3)	0.18940 (15)	0.24922 (14)	0.0400 (6)	
C36	0.8506 (3)	0.13782 (17)	0.32355 (15)	0.0497 (7)	
H36	0.8057	0.0824	0.3349	0.060*	
C37	0.9101 (4)	0.17082 (17)	0.38024 (15)	0.0499 (7)	
C38	0.9792 (3)	0.25185 (17)	0.36690 (15)	0.0490 (7)	
H38	1.0194	0.2716	0.4063	0.059*	
C39	1.0643 (4)	0.39134 (17)	0.27698 (17)	0.0526 (7)	
C40	0.7929 (3)	0.15776 (16)	0.18499 (15)	0.0442 (6)	

### Atomic displacement parameters (Å^2^)

**Table:**

	*U*^11^	*U*^22^	*U*^33^	*U*^12^	*U*^13^	*U*^23^
Co1	0.0385 (2)	0.0391 (2)	0.0386 (2)	−0.00531 (14)	0.00514 (14)	−0.01071 (15)
O1	0.0373 (11)	0.1106 (18)	0.0502 (11)	−0.0070 (11)	0.0047 (9)	0.0014 (11)
O2	0.0393 (10)	0.0708 (13)	0.0427 (10)	−0.0064 (9)	0.0047 (8)	−0.0113 (9)
O3	0.0879 (16)	0.0578 (13)	0.0753 (14)	0.0140 (11)	0.0214 (12)	−0.0227 (11)
O4	0.122 (2)	0.0407 (12)	0.0971 (17)	0.0092 (13)	0.0482 (15)	−0.0090 (12)
O5	0.0532 (11)	0.0453 (10)	0.0435 (10)	−0.0056 (8)	0.0115 (8)	−0.0058 (9)
O6	0.0667 (13)	0.0356 (10)	0.0636 (12)	−0.0036 (9)	0.0135 (10)	−0.0186 (9)
O7	0.197 (3)	0.0594 (17)	0.105 (2)	0.0189 (18)	0.081 (2)	0.0223 (15)
O8	0.141 (2)	0.105 (2)	0.0485 (13)	0.0234 (17)	0.0284 (14)	0.0012 (13)
O9	0.0566 (12)	0.0688 (13)	0.0479 (11)	−0.0180 (10)	0.0014 (9)	−0.0090 (10)
O10	0.0648 (13)	0.0362 (11)	0.0874 (14)	−0.0081 (9)	−0.0061 (11)	−0.0140 (10)
O11	0.155 (3)	0.095 (2)	0.0825 (18)	−0.050 (2)	−0.0184 (17)	0.0320 (15)
O12	0.134 (2)	0.0888 (18)	0.0489 (13)	0.0098 (16)	0.0052 (14)	−0.0129 (13)
O13	0.113 (2)	0.0464 (12)	0.0803 (15)	−0.0072 (12)	−0.0306 (14)	−0.0186 (11)
O14	0.0987 (17)	0.0460 (12)	0.0657 (13)	−0.0224 (12)	−0.0148 (12)	−0.0059 (10)
N1	0.0454 (13)	0.0440 (12)	0.0467 (13)	−0.0119 (10)	0.0052 (10)	−0.0173 (10)
N2	0.0423 (12)	0.0380 (12)	0.0401 (11)	−0.0030 (9)	0.0062 (9)	−0.0113 (9)
N3	0.0472 (13)	0.0427 (12)	0.0395 (12)	0.0007 (10)	0.0047 (9)	−0.0134 (10)
N4	0.0466 (13)	0.0363 (12)	0.0416 (12)	−0.0001 (9)	0.0017 (9)	−0.0058 (9)
N5	0.0764 (17)	0.0393 (13)	0.0539 (14)	−0.0019 (12)	−0.0054 (12)	−0.0002 (11)
N6	0.0599 (16)	0.0653 (16)	0.0443 (13)	−0.0069 (12)	0.0016 (11)	−0.0082 (12)
N7	0.0479 (13)	0.0421 (12)	0.0423 (12)	0.0003 (10)	0.0026 (10)	−0.0155 (10)
N8	0.0449 (12)	0.0374 (12)	0.0410 (11)	−0.0064 (9)	0.0028 (9)	−0.0144 (9)
N9	0.0488 (13)	0.0406 (12)	0.0387 (11)	−0.0074 (10)	0.0018 (10)	−0.0151 (10)
N10	0.0480 (13)	0.0378 (12)	0.0371 (11)	−0.0074 (9)	0.0005 (9)	−0.0062 (9)
N11	0.0755 (17)	0.0397 (13)	0.0511 (13)	−0.0097 (11)	0.0071 (12)	−0.0026 (10)
N12	0.0663 (16)	0.0633 (16)	0.0439 (13)	−0.0107 (13)	0.0083 (12)	−0.0098 (12)
N13	0.092 (2)	0.070 (2)	0.0559 (17)	0.0133 (16)	0.0256 (15)	0.0075 (15)
N14	0.0735 (19)	0.0643 (18)	0.0576 (17)	0.0040 (15)	0.0055 (14)	0.0029 (15)
C1	0.0662 (19)	0.0500 (17)	0.0531 (16)	−0.0129 (14)	0.0030 (14)	−0.0189 (14)
C2	0.074 (2)	0.0546 (18)	0.068 (2)	−0.0172 (15)	0.0070 (16)	−0.0311 (16)
C3	0.083 (2)	0.0444 (17)	0.080 (2)	−0.0195 (15)	0.0135 (18)	−0.0247 (16)
C4	0.074 (2)	0.0392 (16)	0.0591 (18)	−0.0116 (14)	0.0092 (15)	−0.0109 (14)
C5	0.0396 (14)	0.0415 (14)	0.0460 (15)	−0.0048 (11)	0.0060 (11)	−0.0111 (12)
C6	0.0391 (14)	0.0397 (15)	0.0388 (14)	−0.0050 (11)	0.0064 (11)	−0.0077 (11)
C7	0.0349 (14)	0.0445 (15)	0.0414 (14)	0.0002 (11)	0.0058 (11)	−0.0101 (12)
C8	0.0374 (14)	0.0541 (16)	0.0419 (14)	0.0035 (12)	0.0014 (11)	−0.0146 (13)
C9	0.074 (2)	0.088 (2)	0.0420 (17)	−0.0070 (18)	0.0019 (15)	−0.0108 (16)
C10	0.071 (2)	0.098 (3)	0.0492 (18)	0.0009 (19)	−0.0023 (16)	−0.0302 (19)
C11	0.077 (2)	0.073 (2)	0.068 (2)	0.0024 (17)	−0.0076 (17)	−0.0340 (18)
C12	0.0595 (18)	0.0546 (18)	0.0511 (17)	0.0014 (14)	−0.0006 (14)	−0.0169 (14)
C13	0.0656 (19)	0.0552 (18)	0.0498 (16)	0.0038 (14)	0.0043 (14)	−0.0216 (14)
C14	0.074 (2)	0.0540 (18)	0.067 (2)	0.0111 (15)	−0.0033 (16)	−0.0349 (16)
C15	0.082 (2)	0.0390 (16)	0.071 (2)	0.0111 (15)	−0.0118 (17)	−0.0187 (15)
C16	0.069 (2)	0.0420 (16)	0.0523 (16)	0.0030 (14)	−0.0062 (14)	−0.0115 (13)
C17	0.0381 (14)	0.0411 (14)	0.0454 (15)	0.0000 (11)	−0.0023 (11)	−0.0142 (12)
C18	0.0404 (14)	0.0396 (14)	0.0389 (14)	−0.0032 (11)	−0.0020 (11)	−0.0095 (11)
C19	0.0380 (14)	0.0434 (15)	0.0382 (14)	−0.0061 (11)	−0.0007 (11)	−0.0117 (12)
C20	0.0416 (15)	0.0551 (17)	0.0385 (14)	−0.0102 (12)	−0.0002 (11)	−0.0116 (13)
C21	0.079 (2)	0.087 (3)	0.0449 (17)	−0.0169 (19)	0.0120 (16)	−0.0091 (17)
C22	0.066 (2)	0.110 (3)	0.0492 (19)	−0.013 (2)	0.0142 (15)	−0.034 (2)
C23	0.060 (2)	0.085 (2)	0.0607 (19)	0.0022 (17)	0.0004 (15)	−0.0391 (18)
C24	0.0538 (17)	0.0599 (18)	0.0487 (16)	−0.0024 (14)	−0.0030 (13)	−0.0199 (14)
C25	0.0425 (15)	0.0389 (14)	0.0508 (15)	−0.0013 (11)	0.0007 (12)	−0.0151 (12)
C26	0.0399 (14)	0.0364 (14)	0.0452 (14)	−0.0047 (11)	0.0022 (11)	−0.0074 (11)
C27	0.0322 (13)	0.0359 (13)	0.0412 (13)	−0.0033 (10)	−0.0007 (10)	−0.0114 (11)
C28	0.0463 (16)	0.0382 (14)	0.0468 (15)	−0.0011 (12)	0.0014 (12)	−0.0056 (12)
C29	0.0497 (16)	0.0494 (16)	0.0408 (15)	0.0016 (13)	0.0039 (12)	−0.0017 (12)
C30	0.0459 (16)	0.0568 (17)	0.0460 (15)	0.0031 (13)	0.0048 (12)	−0.0169 (13)
C31	0.0594 (19)	0.0441 (16)	0.0625 (18)	−0.0008 (14)	0.0073 (15)	−0.0192 (14)
C32	0.0352 (14)	0.0392 (15)	0.0448 (15)	−0.0085 (11)	0.0008 (11)	−0.0151 (12)
C33	0.0447 (15)	0.0371 (14)	0.0511 (16)	0.0024 (11)	0.0018 (12)	−0.0122 (12)
C34	0.0413 (15)	0.0369 (14)	0.0474 (15)	−0.0004 (11)	0.0031 (12)	−0.0056 (12)
C35	0.0346 (14)	0.0342 (13)	0.0483 (15)	0.0005 (10)	0.0033 (11)	−0.0070 (11)
C36	0.0415 (15)	0.0398 (15)	0.0613 (18)	0.0010 (12)	0.0068 (13)	−0.0042 (13)
C37	0.0493 (17)	0.0480 (16)	0.0453 (16)	0.0087 (13)	0.0049 (13)	−0.0024 (13)
C38	0.0498 (17)	0.0485 (16)	0.0492 (16)	0.0051 (13)	0.0021 (13)	−0.0155 (13)
C39	0.0609 (19)	0.0397 (15)	0.0592 (18)	0.0020 (13)	−0.0052 (15)	−0.0164 (14)
C40	0.0320 (14)	0.0421 (15)	0.0592 (17)	0.0023 (11)	0.0040 (12)	−0.0162 (14)

### Geometric parameters (Å, °)

**Table:**

Co1—O2	2.0548 (18)	C3—C4	1.381 (4)
Co1—O1	2.073 (2)	C3—H3	0.9300
Co1—N2	2.104 (2)	C4—C5	1.374 (3)
Co1—N8	2.114 (2)	C4—H4A	0.9300
Co1—N7	2.151 (2)	C5—C6	1.473 (3)
Co1—N1	2.152 (2)	C7—C8	1.472 (3)
O1—H1A	0.8500	C8—C12	1.383 (4)
O1—H1B	0.8500	C9—C10	1.369 (4)
O2—H2A	0.8500	C9—H9	0.9300
O2—H2B	0.8501	C10—C11	1.367 (4)
O3—C31	1.200 (3)	C10—H10	0.9300
O4—C31	1.320 (3)	C11—C12	1.386 (4)
O4—H4	0.8200	C11—H11	0.9300
O5—C32	1.254 (3)	C12—H12	0.9300
O6—C32	1.251 (3)	C13—C14	1.375 (4)
O7—N13	1.217 (3)	C13—H13	0.9300
O8—N13	1.207 (3)	C14—C15	1.370 (4)
O9—C40	1.251 (3)	C14—H14A	0.9300
O10—C40	1.251 (3)	C15—C16	1.383 (4)
O11—N14	1.211 (3)	C15—H15	0.9300
O12—N14	1.216 (3)	C16—C17	1.374 (3)
O13—C39	1.201 (3)	C16—H16	0.9300
O14—C39	1.315 (3)	C17—C18	1.466 (3)
O14—H14	0.8200	C19—C20	1.462 (3)
N1—C1	1.338 (3)	C20—C24	1.384 (4)
N1—C5	1.357 (3)	C21—C22	1.369 (4)
N2—C6	1.314 (3)	C21—H21	0.9300
N2—N3	1.377 (3)	C22—C23	1.366 (4)
N3—C7	1.317 (3)	C22—H22	0.9300
N4—C6	1.356 (3)	C23—C24	1.377 (4)
N4—C7	1.372 (3)	C23—H23	0.9300
N4—N5	1.419 (3)	C24—H24	0.9300
N5—H5A	0.8897	C25—C30	1.383 (3)
N5—H5B	0.8900	C25—C26	1.389 (3)
N6—C8	1.337 (3)	C25—C31	1.487 (4)
N6—C9	1.340 (3)	C26—C27	1.390 (3)
N7—C13	1.338 (3)	C26—H26	0.9300
N7—C17	1.348 (3)	C27—C28	1.380 (3)
N8—C18	1.314 (3)	C27—C32	1.514 (3)
N8—N9	1.376 (3)	C28—C29	1.383 (3)
N9—C19	1.317 (3)	C28—H28	0.9300
N10—C18	1.352 (3)	C29—C30	1.373 (4)
N10—C19	1.376 (3)	C30—H30	0.9300
N10—N11	1.420 (3)	C33—C34	1.387 (3)
N11—H11A	0.8901	C33—C38	1.388 (3)
N11—H11B	0.8901	C33—C39	1.497 (4)
N12—C20	1.333 (3)	C34—C35	1.389 (3)
N12—C21	1.345 (4)	C34—H34	0.9300
N13—C29	1.474 (3)	C35—C36	1.391 (3)
N14—C37	1.483 (3)	C35—C40	1.514 (3)
C1—C2	1.382 (4)	C36—C37	1.384 (4)
C1—H1	0.9300	C36—H36	0.9300
C2—C3	1.375 (4)	C37—C38	1.374 (4)
C2—H2	0.9300	C38—H38	0.9300
			
O2—Co1—O1	179.70 (7)	C12—C11—H11	120.5
O2—Co1—N2	87.88 (7)	C8—C12—C11	118.2 (3)
O1—Co1—N2	91.82 (7)	C8—C12—H12	120.9
O2—Co1—N8	86.02 (7)	C11—C12—H12	120.9
O1—Co1—N8	94.28 (7)	N7—C13—C14	122.8 (3)
N2—Co1—N8	173.90 (8)	N7—C13—H13	118.6
O2—Co1—N7	94.15 (7)	C14—C13—H13	118.6
O1—Co1—N7	85.93 (8)	C15—C14—C13	118.7 (3)
N2—Co1—N7	103.73 (7)	C15—C14—H14A	120.6
N8—Co1—N7	76.81 (7)	C13—C14—H14A	120.6
O2—Co1—N1	92.39 (7)	C14—C15—C16	119.5 (3)
O1—Co1—N1	87.53 (8)	C14—C15—H15	120.3
N2—Co1—N1	76.85 (8)	C16—C15—H15	120.3
N8—Co1—N1	103.33 (8)	C17—C16—C15	118.6 (3)
N7—Co1—N1	173.44 (8)	C17—C16—H16	120.7
Co1—O1—H1A	119.9	C15—C16—H16	120.7
Co1—O1—H1B	120.3	N7—C17—C16	122.4 (2)
H1A—O1—H1B	116.5	N7—C17—C18	111.6 (2)
Co1—O2—H2A	121.8	C16—C17—C18	126.0 (2)
Co1—O2—H2B	122.6	N8—C18—N10	109.1 (2)
H2A—O2—H2B	115.6	N8—C18—C17	121.0 (2)
C31—O4—H4	109.5	N10—C18—C17	129.8 (2)
C39—O14—H14	109.5	N9—C19—N10	109.8 (2)
C1—N1—C5	117.9 (2)	N9—C19—C20	124.9 (2)
C1—N1—Co1	125.52 (18)	N10—C19—C20	125.3 (2)
C5—N1—Co1	116.25 (16)	N12—C20—C24	123.4 (2)
C6—N2—N3	108.79 (19)	N12—C20—C19	116.6 (2)
C6—N2—Co1	114.47 (15)	C24—C20—C19	120.0 (2)
N3—N2—Co1	135.76 (15)	N12—C21—C22	123.1 (3)
C7—N3—N2	106.38 (19)	N12—C21—H21	118.4
C6—N4—C7	105.5 (2)	C22—C21—H21	118.4
C6—N4—N5	124.6 (2)	C23—C22—C21	119.1 (3)
C7—N4—N5	129.4 (2)	C23—C22—H22	120.5
N4—N5—H5A	103.5	C21—C22—H22	120.5
N4—N5—H5B	102.5	C22—C23—C24	119.3 (3)
H5A—N5—H5B	109.0	C22—C23—H23	120.4
C8—N6—C9	116.7 (3)	C24—C23—H23	120.4
C13—N7—C17	117.9 (2)	C23—C24—C20	118.1 (3)
C13—N7—Co1	125.56 (18)	C23—C24—H24	121.0
C17—N7—Co1	116.28 (15)	C20—C24—H24	121.0
C18—N8—N9	108.96 (18)	C30—C25—C26	119.3 (2)
C18—N8—Co1	113.36 (15)	C30—C25—C31	118.9 (2)
N9—N8—Co1	134.49 (15)	C26—C25—C31	121.7 (2)
C19—N9—N8	106.41 (19)	C25—C26—C27	121.4 (2)
C18—N10—C19	105.68 (19)	C25—C26—H26	119.3
C18—N10—N11	124.7 (2)	C27—C26—H26	119.3
C19—N10—N11	129.2 (2)	C28—C27—C26	119.2 (2)
N10—N11—H11A	103.6	C28—C27—C32	121.0 (2)
N10—N11—H11B	104.0	C26—C27—C32	119.9 (2)
H11A—N11—H11B	108.5	C27—C28—C29	118.6 (2)
C20—N12—C21	116.9 (3)	C27—C28—H28	120.7
O8—N13—O7	124.2 (3)	C29—C28—H28	120.7
O8—N13—C29	118.4 (3)	C30—C29—C28	122.8 (2)
O7—N13—C29	117.4 (3)	C30—C29—N13	118.8 (2)
O11—N14—O12	124.2 (3)	C28—C29—N13	118.3 (2)
O11—N14—C37	117.5 (3)	C29—C30—C25	118.6 (2)
O12—N14—C37	118.3 (3)	C29—C30—H30	120.7
N1—C1—C2	123.1 (3)	C25—C30—H30	120.7
N1—C1—H1	118.4	O3—C31—O4	123.7 (3)
C2—C1—H1	118.4	O3—C31—C25	123.9 (3)
C3—C2—C1	118.1 (3)	O4—C31—C25	112.3 (2)
C3—C2—H2	121.0	O6—C32—O5	124.6 (2)
C1—C2—H2	121.0	O6—C32—C27	118.5 (2)
C2—C3—C4	120.0 (3)	O5—C32—C27	116.9 (2)
C2—C3—H3	120.0	C34—C33—C38	119.8 (2)
C4—C3—H3	120.0	C34—C33—C39	121.6 (2)
C5—C4—C3	118.8 (3)	C38—C33—C39	118.6 (2)
C5—C4—H4A	120.6	C33—C34—C35	121.7 (2)
C3—C4—H4A	120.6	C33—C34—H34	119.1
N1—C5—C4	122.1 (2)	C35—C34—H34	119.1
N1—C5—C6	111.4 (2)	C34—C35—C36	118.7 (2)
C4—C5—C6	126.4 (2)	C34—C35—C40	120.3 (2)
N2—C6—N4	109.2 (2)	C36—C35—C40	121.1 (2)
N2—C6—C5	120.5 (2)	C37—C36—C35	118.5 (2)
N4—C6—C5	130.2 (2)	C37—C36—H36	120.7
N3—C7—N4	110.0 (2)	C35—C36—H36	120.7
N3—C7—C8	124.2 (2)	C38—C37—C36	123.4 (2)
N4—C7—C8	125.8 (2)	C38—C37—N14	117.8 (3)
N6—C8—C12	123.4 (2)	C36—C37—N14	118.8 (3)
N6—C8—C7	116.7 (2)	C37—C38—C33	117.9 (3)
C12—C8—C7	119.9 (2)	C37—C38—H38	121.1
N6—C9—C10	123.8 (3)	C33—C38—H38	121.1
N6—C9—H9	118.1	O13—C39—O14	123.3 (3)
C10—C9—H9	118.1	O13—C39—C33	123.7 (3)
C11—C10—C9	118.8 (3)	O14—C39—C33	112.9 (2)
C11—C10—H10	120.6	O9—C40—O10	124.7 (3)
C9—C10—H10	120.6	O9—C40—C35	117.1 (2)
C10—C11—C12	119.1 (3)	O10—C40—C35	118.1 (2)
C10—C11—H11	120.5		
			
O2—Co1—N1—C1	−97.4 (2)	Co1—N7—C17—C16	174.8 (2)
O1—Co1—N1—C1	82.9 (2)	C13—N7—C17—C18	−178.6 (2)
N2—Co1—N1—C1	175.3 (2)	Co1—N7—C17—C18	−3.9 (3)
N8—Co1—N1—C1	−10.9 (2)	C15—C16—C17—N7	0.2 (4)
O2—Co1—N1—C5	89.02 (18)	C15—C16—C17—C18	178.7 (3)
O1—Co1—N1—C5	−90.69 (18)	N9—N8—C18—N10	1.1 (3)
N2—Co1—N1—C5	1.76 (17)	Co1—N8—C18—N10	163.97 (15)
N8—Co1—N1—C5	175.50 (17)	N9—N8—C18—C17	−174.5 (2)
O2—Co1—N2—C6	−90.27 (17)	Co1—N8—C18—C17	−11.7 (3)
O1—Co1—N2—C6	89.72 (18)	C19—N10—C18—N8	−1.5 (3)
N7—Co1—N2—C6	175.97 (17)	N11—N10—C18—N8	−175.0 (2)
N1—Co1—N2—C6	2.69 (17)	C19—N10—C18—C17	173.7 (2)
O2—Co1—N2—N3	76.8 (2)	N11—N10—C18—C17	0.2 (4)
O1—Co1—N2—N3	−103.2 (2)	N7—C17—C18—N8	10.6 (3)
N7—Co1—N2—N3	−17.0 (2)	C16—C17—C18—N8	−168.1 (3)
N1—Co1—N2—N3	169.7 (2)	N7—C17—C18—N10	−164.1 (2)
C6—N2—N3—C7	0.3 (2)	C16—C17—C18—N10	17.3 (4)
Co1—N2—N3—C7	−167.25 (18)	N8—N9—C19—N10	−0.6 (3)
O2—Co1—N7—C13	−102.1 (2)	N8—N9—C19—C20	176.3 (2)
O1—Co1—N7—C13	77.6 (2)	C18—N10—C19—N9	1.3 (3)
N2—Co1—N7—C13	−13.2 (2)	N11—N10—C19—N9	174.4 (2)
N8—Co1—N7—C13	173.0 (2)	C18—N10—C19—C20	−175.6 (2)
O2—Co1—N7—C17	83.76 (18)	N11—N10—C19—C20	−2.5 (4)
O1—Co1—N7—C17	−96.53 (18)	C21—N12—C20—C24	−2.7 (4)
N2—Co1—N7—C17	172.59 (17)	C21—N12—C20—C19	176.7 (2)
N8—Co1—N7—C17	−1.16 (17)	N9—C19—C20—N12	164.5 (2)
O2—Co1—N8—C18	−88.54 (17)	N10—C19—C20—N12	−19.0 (4)
O1—Co1—N8—C18	91.47 (18)	N9—C19—C20—C24	−16.0 (4)
N7—Co1—N8—C18	6.67 (17)	N10—C19—C20—C24	160.4 (2)
N1—Co1—N8—C18	179.93 (17)	C20—N12—C21—C22	0.8 (5)
O2—Co1—N8—N9	68.4 (2)	N12—C21—C22—C23	1.3 (5)
O1—Co1—N8—N9	−111.5 (2)	C21—C22—C23—C24	−1.6 (5)
N7—Co1—N8—N9	163.7 (2)	C22—C23—C24—C20	−0.2 (4)
N1—Co1—N8—N9	−23.1 (2)	N12—C20—C24—C23	2.5 (4)
C18—N8—N9—C19	−0.3 (3)	C19—C20—C24—C23	−176.9 (2)
Co1—N8—N9—C19	−158.00 (18)	C30—C25—C26—C27	−0.2 (4)
C5—N1—C1—C2	−0.5 (4)	C31—C25—C26—C27	177.5 (2)
Co1—N1—C1—C2	−174.0 (2)	C25—C26—C27—C28	−0.9 (4)
N1—C1—C2—C3	0.9 (5)	C25—C26—C27—C32	178.9 (2)
C1—C2—C3—C4	−0.5 (5)	C26—C27—C28—C29	0.7 (4)
C2—C3—C4—C5	−0.2 (5)	C32—C27—C28—C29	−179.1 (2)
C1—N1—C5—C4	−0.3 (4)	C27—C28—C29—C30	0.6 (4)
Co1—N1—C5—C4	173.8 (2)	C27—C28—C29—N13	−177.8 (2)
C1—N1—C5—C6	−179.4 (2)	O8—N13—C29—C30	12.9 (5)
Co1—N1—C5—C6	−5.3 (3)	O7—N13—C29—C30	−163.9 (3)
C3—C4—C5—N1	0.7 (4)	O8—N13—C29—C28	−168.7 (3)
C3—C4—C5—C6	179.6 (3)	O7—N13—C29—C28	14.5 (5)
N3—N2—C6—N4	0.4 (3)	C28—C29—C30—C25	−1.7 (4)
Co1—N2—C6—N4	170.89 (15)	N13—C29—C30—C25	176.7 (3)
N3—N2—C6—C5	−177.3 (2)	C26—C25—C30—C29	1.4 (4)
Co1—N2—C6—C5	−6.8 (3)	C31—C25—C30—C29	−176.4 (2)
C7—N4—C6—N2	−0.9 (3)	C30—C25—C31—O3	7.8 (4)
N5—N4—C6—N2	−174.0 (2)	C26—C25—C31—O3	−169.9 (3)
C7—N4—C6—C5	176.5 (2)	C30—C25—C31—O4	−174.9 (3)
N5—N4—C6—C5	3.4 (4)	C26—C25—C31—O4	7.3 (4)
N1—C5—C6—N2	8.1 (3)	C28—C27—C32—O6	1.1 (3)
C4—C5—C6—N2	−170.9 (3)	C26—C27—C32—O6	−178.7 (2)
N1—C5—C6—N4	−169.0 (2)	C28—C27—C32—O5	180.0 (2)
C4—C5—C6—N4	12.0 (4)	C26—C27—C32—O5	0.2 (3)
N2—N3—C7—N4	−0.9 (3)	C38—C33—C34—C35	−0.1 (4)
N2—N3—C7—C8	179.8 (2)	C39—C33—C34—C35	178.5 (2)
C6—N4—C7—N3	1.1 (3)	C33—C34—C35—C36	0.4 (4)
N5—N4—C7—N3	173.8 (2)	C33—C34—C35—C40	179.0 (2)
C6—N4—C7—C8	−179.6 (2)	C34—C35—C36—C37	−0.1 (4)
N5—N4—C7—C8	−7.0 (4)	C40—C35—C36—C37	−178.6 (2)
C9—N6—C8—C12	−0.4 (4)	C35—C36—C37—C38	−0.6 (4)
C9—N6—C8—C7	−178.4 (2)	C35—C36—C37—N14	178.1 (2)
N3—C7—C8—N6	163.6 (2)	O11—N14—C37—C38	176.0 (3)
N4—C7—C8—N6	−15.6 (4)	O12—N14—C37—C38	−4.1 (4)
N3—C7—C8—C12	−14.5 (4)	O11—N14—C37—C36	−2.8 (4)
N4—C7—C8—C12	166.3 (2)	O12—N14—C37—C36	177.2 (3)
C8—N6—C9—C10	1.0 (5)	C36—C37—C38—C33	0.9 (4)
N6—C9—C10—C11	−0.5 (5)	N14—C37—C38—C33	−177.8 (2)
C9—C10—C11—C12	−0.7 (5)	C34—C33—C38—C37	−0.6 (4)
N6—C8—C12—C11	−0.7 (4)	C39—C33—C38—C37	−179.2 (2)
C7—C8—C12—C11	177.3 (2)	C34—C33—C39—O13	−166.1 (3)
C10—C11—C12—C8	1.3 (4)	C38—C33—C39—O13	12.5 (4)
C17—N7—C13—C14	−0.5 (4)	C34—C33—C39—O14	13.0 (4)
Co1—N7—C13—C14	−174.6 (2)	C38—C33—C39—O14	−168.4 (2)
N7—C13—C14—C15	0.5 (5)	C34—C35—C40—O9	2.0 (3)
C13—C14—C15—C16	−0.2 (5)	C36—C35—C40—O9	−179.5 (2)
C14—C15—C16—C17	−0.2 (4)	C34—C35—C40—O10	−175.1 (2)
C13—N7—C17—C16	0.1 (4)	C36—C35—C40—O10	3.4 (3)

### Hydrogen-bond geometry (Å, °)

**Table:**

*D*—H···*A*	*D*—H	H···*A*	*D*···*A*	*D*—H···*A*
N5—H5A···N6	0.89	2.23	2.885 (3)	130
N11—H11A···N12	0.89	2.21	2.859 (3)	130
O1—H1A···O9^i^	0.85	1.85	2.674 (3)	163
O1—H1B···O5^ii^	0.85	1.87	2.710 (3)	168
O2—H2A···O5^iii^	0.85	1.89	2.712 (2)	162
O2—H2B···O9	0.85	1.86	2.681 (3)	163
O4—H4···O10^iv^	0.82	1.73	2.545 (3)	172
N5—H5B···O10	0.89	2.36	3.065 (3)	136
N5—H5B···O3^iv^	0.89	2.51	3.234 (3)	139
N11—H11B···O6^iii^	0.89	2.32	3.057 (3)	140
N11—H11B···O13^v^	0.89	2.56	3.252 (3)	135
O14—H14···O6^vi^	0.82	1.77	2.580 (3)	170
C3—H3···O5^vii^	0.93	2.47	3.402 (4)	176
C9—H9···O11^iv^	0.93	2.49	3.249 (5)	139
C14—H14A···O6^viii^	0.93	2.49	3.403 (4)	166
C21—H21···O7^ix^	0.93	2.48	3.259 (4)	142
C28—H28···O13^vi^	0.93	2.56	3.490 (4)	175

Symmetry codes: (i) *x*−1, *y*, *z*; (ii) *x*, *y*, *z*−1; (iii) *x*+1, *y*, *z*−1; (iv) −*x*+1, −*y*, −*z*+1; (v) −*x*+2, −*y*+1, −*z*; (vi) −*x*+1, −*y*+1, −*z*+1; (vii) −*x*, −*y*, −*z*+1; (viii) −*x*, −*y*+1, −*z*+1; (ix) −*x*+1, −*y*+1, −*z*.
